# Early Decline in Thyroid Hormone Levels Predicts Mortality Following Congenital Heart Surgery in Neonates: A Retrospective Cohort Study

**DOI:** 10.3390/diagnostics16010070

**Published:** 2025-12-25

**Authors:** Duygu Tunçel, Süleyman Geter, Leyla Şero, Nilüfer Okur, Osman Akdeniz

**Affiliations:** 1 Department of Pediatrics, Division of Neonatology, SBU Gazi Yasargil Training and Research Hospital, 21010 Diyarbakır, Turkey; nilufermatur.okur@saglik.com; 2 Department of Pediatrics, SBU Gazi Yasargil Training and Research Hospital, 21010 Diyarbakır, Turkey; sgeter21@gmail.com (S.G.); ozlemleylasero@gmail.com (L.Ş.); 3 Department of Pediatric Cardiyology, SBU Gazi Yaşargil Training and Research Hospital, 21010 Diyarbakır, Turkey; osman_akdeniz@hotmail.com

**Keywords:** congenital heart surgery, FT3, FT4, TSH, neonates, cardiopulmonary bypass

## Abstract

**Background:** Thyroid hormone dysregulation is a well-recognized consequence of cardiopulmonary bypass (CPB), particularly in neonates undergoing congenital heart surgery. Triiodothyronine (T3) plays a crucial role in maintaining cardiovascular stability, and an early decline in serum levels may adversely impact clinical outcomes. This study aimed to evaluate perioperative thyroid hormone changes and their association with morbidity and mortality. **Methods:** We retrospectively analyzed 132 neonates who underwent congenital cardiac surgery with CPB between January 2021 and June 2024. Serum free T3 (FT3), free thyroxine (FT4), and thyroid-stimulating hormone (TSH) levels were measured preoperatively and within one hour after admission to the cardiac intensive care unit. Demographic, clinical, and surgical variables were recorded. Associations between thyroid hormone levels and postoperative outcomes, including in-hospital mortality, ventilation duration, vasoactive-inotropic score (VIS), and length of stay, were assessed using correlation analyses, logistic regression, and receiver operating characteristic (ROC) analysis. **Results:** Postoperatively, both FT3 and TSH levels declined significantly (*p* < 0.01), while FT4 levels remained unchanged. Lower postoperative FT3 levels were negatively correlated with prolonged invasive mechanical ventilation (rho = −0.196, *p* = 0.029) and longer hospital stay (rho = −0.183, *p* = 0.042). Overall mortality was 7.6% (*n* = 10). Non-survivors had significantly lower postoperative FT3 levels compared with survivors (*p* = 0.001). In multivariable logistic regression, postoperative FT3 was independently associated with mortality (OR = 0.22, 95% CI 0.05–1.03, *p* = 0.048). ROC analysis demonstrated good predictive performance of postoperative FT3 for mortality (AUC = 0.818), with an optimal cutoff of 2.17 pg/mL (sensitivity 72%, specificity 70%). **Conclusions:** Early postoperative suppression of FT3 is common after CPB in neonates and is independently associated with increased mortality and adverse short-term outcomes. Early assessment of thyroid function, particularly FT3, may provide valuable prognostic information and aid in risk stratification in this high-risk population.

## 1. Introduction

Thyroid function in neonates with congenital heart disease undergoing cardiopulmonary bypass (CPB) is a critical area of research. Cardiopulmonary bypass affects normal homeostasis, especially during the neonatal period, and has significant effects on thyroid function [1,2]. The thyroid gland plays a vital role in regulating metabolism and cardiovascular function, which is particularly important in the context of cardiac surgery [3].

Following CPB, neonates often experience a transient hypothyroid state, characterized by decreased levels of triiodothyronine (T3) and thyroxine (T4), along with alterations in thyroid-stimulating hormone (TSH) levels. This phenomenon, known as euthyroid sick syndrome or non-thyroidal illness syndrome, is thought to result from the systemic inflammatory response, hemodilution, hypothermia, and stress associated with CPB [2,4]. Disruption of thyroid hormone balance due to CPB may contribute to adverse clinical outcomes, including impaired hemodynamic stability, altered metabolic response, and an increased risk of low cardiac output syndrome (LCOS) [1].

T3 and T4 exert important cardiovascular effects, particularly through modulation of myocardial contractility and systemic vascular resistance. In the postoperative period, decreased serum T3 and T4 levels following cardiopulmonary bypass may increase inotropic requirements and affect hemodynamic stability [1,2,5]. In patients younger than three months, postoperative T3 supplementation has been shown to reduce vasoactive-inotropic scores, suggesting a clinically relevant interaction between thyroid hormone status and cardiovascular support in early life [5].

Altered thyroid function after CPB may therefore influence postoperative recovery, affecting cardiac output, systemic vascular resistance, and overall hemodynamic stability [2,6,7]. Several studies have demonstrated associations between thyroid hormone levels and postoperative outcomes such as duration of mechanical ventilation, length of intensive care unit stay, and mortality [2,6,7]. Notably, a relationship between preoperative free T3 levels and mortality after congenital heart surgery has been reported in infants younger than three months [2].

However, despite these findings, data focusing exclusively on neonates remain limited. Many existing studies include heterogeneous pediatric populations, combining neonates with older infants, which may obscure neonatal-specific physiological responses. In addition, most studies assess thyroid hormone levels at later postoperative time points, while evidence regarding very early postoperative thyroid hormone changes and their prognostic significance in neonates is scarce. Given the limited hormone reserves and immature stress responses in newborns, neonatal-specific evaluations are warranted.

Based on this identified knowledge gap, we hypothesized that perioperative, particularly early postoperative, reductions in T3 and T4 levels in neonates undergoing cardiopulmonary bypass are associated with adverse short-term outcomes, including increased mortality. Accordingly, the primary aim of this study was to investigate the relationship between preoperative and early postoperative thyroid hormone levels (T3, T4, and TSH) and postoperative clinical outcomes in neonates undergoing congenital heart surgery with CPB.

## 2. Materials and Methods

### 2.1. Study Design and Setting

This is a retrospective cohort study conducted in the Neonatal Cardiac Intensive Care Unit (CICU) of the Ministry of Health Gazi Yaşargil Training and Research Hospital, Diyarbakır, Turkey. The study period extended from 1 January 2021, to 30 June 2024. All medical records were reviewed through the institutional electronic patient information system and the hospital archive. Since our study was designed as a retrospective cohort study, an a priori sample size calculation and power analysis were not performed at the beginning of the study.

### 2.2. Participants

**Figure 1:**
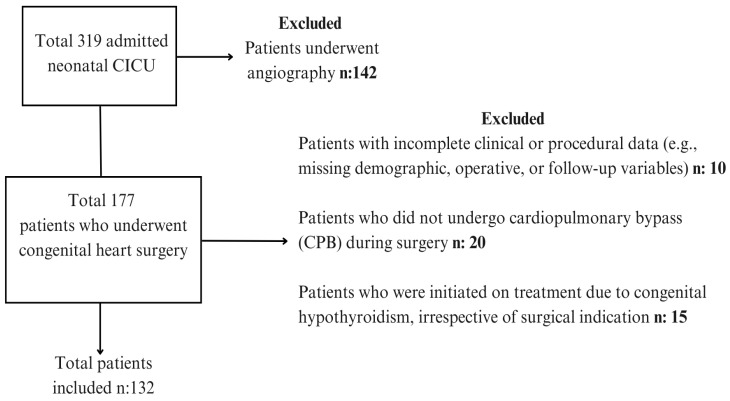
Study Flowchart Depicting Neonatal Patient Inclusion and Exclusion.

All neonates (0–28 days of age) who underwent congenital cardiac surgery with the use of cardiopulmonary bypass (CPB) during the study period were eligible for inclusion. A total of 132 neonates met these criteria. Exclusion criteria were: (1) patients who died before surgery or intraoperatively, and (2) incomplete or missing medical data. The flow of participants is illustrated in Figure 1.

### 2.3. Variables and Data Collection

Baseline demographic data included sex, birth weight, and gestational age. Clinical and perioperative variables comprised cardiac diagnosis, Risk Adjustment for Congenital Heart Surgery Score (RACHS), age at surgery, surgical procedure, duration of CPB and aortic cross-clamp time, vasoactive inotropic score (VIS), initiation of enteral feeding, duration of invasive and non-invasive mechanical ventilation, length of stay in the CICU and hospital, and requirement for extracorporeal therapies such as peritoneal dialysis, hemodialysis, plasma exchange, or extracorporeal membrane oxygenation (ECMO). Mortality during the index hospitalization was recorded as the primary outcome.

All clinical and laboratory data were extracted from the hospital information system by the principal investigator. Thyroid hormone assays were performed in the hospital’s central laboratory using standardized immunoassay methods. Ventilation parameters, inotropic support, and extracorporeal therapies were obtained from daily nursing and physician records. To minimize selection bias, all consecutive eligible neonates during the study period were included. Measurement bias was reduced by using standardized laboratory methods and electronic records.

Data abstraction was performed by one investigator and verified by a second reviewer. The study size was determined by the total number of eligible patients (*n* = 132) within the defined study period; no prior sample size calculation was performed.

### 2.4. Assessment of Thyroid Function and Treatment

Preoperative thyroid function tests (serum free T3, free T4, and thyroid-stimulating hormone [TSH]) were performed in all patients. For neonates admitted to the CICU within the first five days of life, testing was performed on postnatal day 5; for those admitted after day 5, tests were obtained at admission. Postoperative hormone levels were measured one hour after CICU admission following CPB. Thyroid function categories were defined as:

- TSH 0–5 mIU/L: normal; 5–10 mIU/L: mildly elevated; >10 mIU/L: markedly elevated;
- Free T4: normal 1.18–2.49 ng/dL;
- Free T3: normal 1.43–3.26 pg/mL.

Patients with normal values received no therapy. Those with TSH 5–10 mIU/L underwent repeat testing after 7 days. Patients with TSH >10 mIU/L received triiodothyronine supplementation at 10 mcg/kg/day.

During the study period, serum Free T3, Free T4, and TSH levels were measured in all patients in the same central laboratory using the same analytical methods and the same commercial assay kits. In our unit, measurement of serum free T3, free T4, and TSH levels is routinely performed in neonates with CHD admitted to the CICU, as part of standard clinical assessment and follow-up.

### 2.5. Primary and Secondary Outcomes

The primary outcome of this study was to evaluate the association between preoperative and postoperative TSH, T3, and T4 levels and in-hospital mortality in neonates undergoing CPB.

The secondary outcomes included the assessment of the relationships between thyroid hormone levels and the VIS, duration of invasive mechanical ventilation, duration of noninvasive mechanical ventilation, duration of supplemental oxygen therapy, and length of hospital stay.

### 2.6. Statistical Analysis

Statistical analysis was carried out using SPSS version 22.0 (SPSS Inc., Chicago, IL, USA). Continuous variables were tested for normality using the Shapiro–Wilk test and presented as mean ± standard deviation or median (minimum–maximum), as appropriate. Between-group comparisons were performed using Student’s *t*-test or Mann–Whitney U test. Categorical variables were analyzed with the chi-square test or Fisher’s exact test. Correlation analyses were conducted using Pearson or Spearman correlation coefficients. To evaluate the effects of serum thyroid hormone levels on mortality, regression analyses were performed. In addition, receiver operating characteristic (ROC) curve analysis was conducted to assess the predictive ability of serum free T3 levels for mortality. Sensitivity (%), specificity (%), and the optimal cutoff value were calculated. A *p*-value of <0.05 was considered statistically significant.

### 2.7. Ethical Approval

This study was conducted in accordance with the Declaration of Helsinki, and approved by the Ethics Committee of SBU Gazi Yasargil Training and Research Hospital (Protocol code: 2024/186, Date: 12 January 2024). Written informed consent has been obtained from the parents/guardians of all patients to publish this paper.

## 3. Results

### 3.1. Baseline Characteristics

A total of 132 patients who underwent surgery for congenital heart disease were included in the study. Basic demographic characteristics of the study population are summarized in Table 1. The mean birth weight was 2863 ± 493 g, and the mean gestational age was 38 ± 1.4 weeks. Among the participants, 15.2% (*n* = 20) were born preterm, and 54.5% (*n* = 72) were male. The majority of patients (78%, *n* = 103) were delivered via cesarean section. Median postnatal surgery day was 21 (min 8–max 31) (Table 1).

The most common congenital heart defect was transposition of the great arteries, accounting for 37.9% (*n* = 50) of the cases. This was followed by aortic coarctation in 22.7% (*n* = 30), and hypoplastic left heart syndrome, truncus arteriosus, and hypoplasia of the aortic arch, each present in 7.7% (*n* = 10) of patients. Less common diagnoses included tricuspid atresia, pulmonary atresia, total anomalous pulmonary venous return, and aortic stenosis. Rare forms such as Taussig–Bing anomaly, tetralogy of Fallot, and other unclassified congenital heart defects were also observed at lower frequencies (Table 2).

### 3.2. Thyroid Hormone Changes After Surgery

In the preoperative period, 13% of patients had TSH levels above 5 mIU/L, whereas this proportion decreased to 4.5% in the postoperative period. Similarly, 3% of patients had FT3 levels below the lower reference limit of 1.18 ng/dL preoperatively, and this rate increased to 7.5% postoperatively. FT4 levels were below the lower reference threshold of 1.43 pg/mL in 12% of patients in both the preoperative and postoperative periods. Preoperative and postoperative levels of TSH, FT3, and FT4 were compared to assess the impact of surgery on thyroid function. As shown in Table 3, both TSH and FT3 levels significantly decreased in the postoperative period (*p* < 0.01). In contrast, FT4 levels showed no statistically significant change between the preoperative and postoperative measurements (*p* = 0.458).

Analysis of postoperative FT3 levels demonstrated significant negative correlations with selected clinical outcomes. Lower postoperative FT3 levels were associated with prolonged hospital stay (rho = −0.183, *p* = 0.042) and longer invasive mechanical ventilation duration (rho = −0.196, *p* = 0.029). No significant correlations were observed between postoperative T3 levels and VIS, CPB duration, aortic cross-clamp duration, or non-invasive ventilation times (all *p* > 0.05) (Table 4).

Correlation analysis between preoperative and postoperative T4 levels and clinical parameters revealed significant associations in selected variables. Preoperative T4 levels showed a positive correlation with aortic cross-clamp duration (rho = 0.229, *p* = 0.013), whereas postoperative T4 levels did not demonstrate any significant correlation with cross-clamp time (rho = 0.005, *p* = 0.955). Additionally, lower preoperative T4 levels were moderately associated with longer hospital stay (rho = −0.189, *p* = 0.039). No significant correlations were observed between pre- or postoperative T4 levels and postoperative VIS, CPB duration, or invasive/non-invasive mechanical ventilation times (all *p* > 0.05) (Table 5).

### 3.3. Mortality Analysis

A total of 10 patients (7.6%) died. Four patients died due to sepsis-related multiorgan failure, two due to necrotizing enterocolitis, and four due to low cardiac output syndrome and capillary leak syndrome. As shown in Table 6, non-survivors had significantly lower preoperative TSH levels and postoperative FT3 levels compared with survivors (*p* = 0.006 and *p* = 0.001, respectively). In addition, non-survivors exhibited significantly higher preoperative VIS and markedly longer durations of mechanical ventilation (*p* = 0.002 and *p* < 0.001, respectively). Postoperative TSH and preoperative FT3 levels showed a trend toward lower values in the non-survivor group; however, these differences did not reach statistical significance. No significant differences were observed between survivors and non-survivors with respect to preoperative or postoperative FT4 levels, postoperative VIS, operation day, aortic cross-clamp time, cardiopulmonary bypass duration, or RACHS score.

Table 7 presents the results of the logistic regression analysis evaluating factors associated with mortality. Among the variables examined, postoperative FT3 level was the only factor significantly associated with mortality, with lower postoperative FT3 levels corresponding to an increased risk of death (OR = 0.22, 95% CI = 0.05–1.03, *p* = 0.048). Although higher RACHS scores and preoperative VIS showed a tendency toward increased mortality risk, these associations did not reach statistical significance. Similarly, cardiopulmonary bypass duration, postoperative VIS, gestational age, birth weight, preoperative TSH, and postoperative FT4 levels were not significantly associated with mortality.

**Figure 2:**
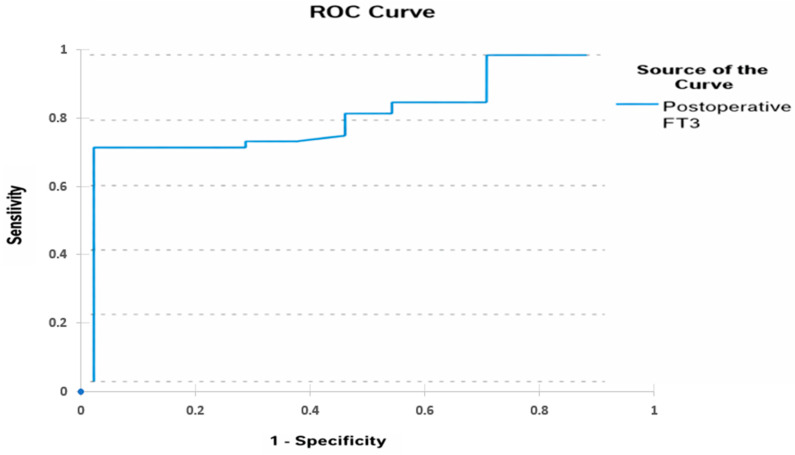
ROCcurve of the postoperative FT3 predicting mortality.

ROC curve analysis revealed that postoperative FT3 had good discriminatory ability for predicting mortality, with an area under the curve (AUC) of 0.818 (95% CI: 0.732–0.905, *p* < 0.01). An optimal cut-off value of 2.17 for postoperative FT3 yielded a sensitivity of 72% and a specificity of 70% (Figure 2, Table 8).

## 4. Discussion

Early postoperative declines in FT3 levels strongly predicted in-hospital mortality in neonates undergoing cardiopulmonary bypass (CPB). In this retrospective cohort, neonates who died during hospitalization had significantly lower FT3 levels measured within the first postoperative hour. Postoperative FT3 emerged as the only independent predictor of mortality in multivariable analysis. These findings identify early FT3 suppression as a powerful prognostic biomarker in this highly vulnerable population.

Previous studies have shown that thyroid hormone alterations after cardiac surgery with CPB—particularly reductions in FT3 and other thyroid parameters—occur frequently in infants and children undergoing corrective cardiac procedures [4]. This pattern is commonly associated with non-thyroidal illness syndrome. Multiple mechanisms contribute, including systemic inflammation, cytokine release, altered deiodinase activity, hypothermia, hemodilution, and suppression of the hypothalamic–pituitary–thyroid axis [4]. Among these changes, reduced T3 levels are considered a key biochemical marker reflecting impaired metabolic and cardiovascular adaptation during critical illness.

Earlier investigations in infants and children with congenital heart disease (CHD) reported significant postoperative decreases in FT3, FT4, and TSH levels, with only partial recovery within the first 24–72 h [4]. This biphasic hormonal response becomes more pronounced as surgical complexity and systemic stress increase. Talwar et al. demonstrated reductions exceeding 45% in FT3 and FT4 within the first 24 postoperative hours. They also showed that a lower area under the curve (AUC) for total T4 was associated with mortality and a complicated postoperative course [3].

One of the most distinctive aspects of the present study is the timing of thyroid hormone measurements. We measured FT3, FT4, and TSH levels within the first hour after admission to the neonatal cardiac intensive care unit following CPB. Most previous studies focused on measurements obtained 24–48 h after surgery. By capturing the immediate postoperative hormonal suppression, our approach provides early prognostic information. This very early window is clinically critical for identifying neonates at high risk of hemodynamic instability [8].

In our cohort, early postoperative FT3 levels showed a strong and independent association with mortality. In multivariable models that included established risk factors such as RACHS score, CPB duration, and vasoactive–inotropic score, only FT3 remained statistically significant. This finding suggests that FT3 is not merely a surrogate marker of disease severity. Instead, it may reflect inadequate global physiological adaptation to surgical stress. Consistent with our results, Baysal et al. reported that low T3 levels after congenital heart surgery were associated with low cardiac output syndrome and poor prognosis [9].

Mortality in our cohort was associated with severe clinical conditions characterized by systemic inflammation and hemodynamic instability, including low cardiac output syndrome, capillary leak syndrome, sepsis, and necrotizing enterocolitis. Thyroid hormones—particularly T3—directly influence myocardial contractility, vascular tone, and cellular metabolism. Therefore, early FT3 suppression may contribute to impaired cardiovascular performance and increased susceptibility to multiorgan dysfunction. Although causality cannot be established because of the retrospective design, the consistent association between low FT3 levels and adverse outcomes appears biologically plausible [10].

In addition, low early postoperative FT3 levels were associated with prolonged invasive mechanical ventilation and longer hospital stays. These findings support the clinical relevance of FT3 not only for mortality prediction but also for short-term morbidity, recovery time, and intensive care resource utilization.

Preoperative data further support the prognostic role of FT3. Yu et al. identified low preoperative FT3 levels as an independent predictor of intensive care unit mortality in neonates with CHD and reported an AUC of 0.856 for FT3 [5]. In our study, the AUC for postoperative FT3 was 0.818. The identified cutoff value showed moderate sensitivity (72%) and specificity (70%). Together, these results reinforce FT3 as a strong discriminative biomarker.

Beyond observational evidence, interventional studies have evaluated the therapeutic potential of T3 replacement [1,2,5,11,12]. In a randomized controlled trial, Karri et al. demonstrated that oral T3 administration after CPB reduced vasoactive–inotropic scores and improved oxygenation indices, particularly in infants younger than six months undergoing complex cardiac surgery [13]. Although we did not assess hormone replacement therapy, the strong association between low FT3 levels and adverse outcomes underscores the need for targeted interventional trials in high-risk neonates.

The main strengths of this study include very early postoperative measurement of thyroid hormones in neonates undergoing CPB, concurrent evaluation of clinically meaningful mortality and morbidity outcomes, and adjustment for potential confounders using multivariable analyses. Several limitations should be acknowledged. The retrospective, single-center design and relatively small sample size limit generalizability. Thyroid hormone measurements were confined to the early postoperative period. In addition, non-standardized intraoperative practices and postoperative management strategies may have influenced outcomes.

## 5. Conclusions

In conclusion, early postoperative FT3 suppression is a strong and independent predictor of mortality and morbidity in neonates undergoing CPB. Routine early assessment of FT3 in neonatal cardiac intensive care units may facilitate early risk stratification and support individualized postoperative management strategies.

## Figures and Tables

**Table 1:** Basic demographic characteristics of the participants.

	N = 132
Birth weight, g *	2863 ± 493
Gestational age, weeks *	38 ± 1.4
Prematurity (<37 Gw), *n* (%)	20 (15.2)
Male, *n* (%)	72 (54.5)
C/S, *n* (%)	103 (78)
Postnatal age at surgery (days) **	21 (8–31)

* Mean ± Std deviation; ** Median (minimum-maximum); C/S: Sectio caesarea.

**Table 2:** The primary diagnoses of the patients.

	N (132)	Percent
Hypoplastic left heart syndrome	10	7.7
Transposition of the great arteries	50	37.9
Truncus arteriosus	10	7.7
Aortic coarctation	30	22.7
Hypoplasia of the aortic arch	10	7.7
Tricuspid atresia	4	3
Pulmonary atresia	4	3
Taussig–Bing anomaly	2	1.5
Total pulmonary venous return anomaly	4	3
Tetralogy of Fallot	2	1.5
Aortic stenosis	4	3
Others	2	1.5

**Table 3:** Comparison of Preoperative and Postoperative Levels of TSH, FT3, and FT4.

		Preoperative	Postoperative	*p*
TSH mIU/L	Mean ± Std dev	5.4 ± 5.6	3.06 ± 5.3	
	Median (Min–Max)	4.1 (0.03–37.7)	1.6 (0.09–39)	<0.01
FT3 pg/mL	Mean ± Std dev	2.8 ± 1.1	2.39 ± 0.86	<0.01
	Median (Min–Max)	2.61 (1.11–6.1)	2.2 (1.13–4.72)	
FT4 ng/dL	Mean ± Std dev	1.52 ±0.31	1.49 ± 0.39	
	Median (Min–Max)	1.54 (0.91–2.91)	1.48 (0.19–3.23)	0.458

FT3: Free triiodothyronine, FT4: Free thyroxine, TSH: thyroid-stimulating hormone.

**Table 4:** Correlation Between Postoperative FT3 Levels and Clinical Parameters.

Parameter	Spearman’s Rho	*p*-Value	Significance
Postoperative VIS	−0.003	0.978	Not significant
CPB duration (minutes)	−0.108	0.247	Not significant
Aortic cross-clamp duration (minutes)	−0.015	0.867	Not significant
Length of hospital stay (days)	−0.183	0.042	* Significant (*p* < 0.05)
Invasive mechanical ventilation duration (days)	−0.196	0.029	* Significant (*p* < 0.05)
Non-invasive mechanical ventilation duration (days)	−0.114	0.221	Not significant

FT3: Free triiodothyronine, VIS: Vasoactive Inotropic Score, CPB: Cardiopulmonary Bypass. * ** *p* < 0.05 was considered statistically significant.

**Table 5:** Correlation Between Preoperative and Postoperative FT4 Levels and Clinical Parameters.

Parameter	Preop FT4 (Rho)	Preop FT4 (*p*)	Postop FT4 (Rho)	PostopF T4 (*p*)
Postoperative VIS	0.096	0.295	0.042	0.643
CPB duration (minutes)	0.134	0.156	−0.068	0.471
Aortic cross-clamp duration (minutes)	0.229	0.013	0.005	0.955
Length of hospital stay (days)	−0.189	0.039	−0.139	0.126
Invasive mechanical ventilation duration (days)	0.013	0.889	−0.001	0.989
Non-invasive mechanical ventilation duration	−0.022	0.821	0.017	0.859

FT4: Free thyroxine, VIS: Vasoactive Inotropic Score, CPB: Cardiopulmonary Bypass.

**Table 6:** Comparison of survivors and non-survivors according to mortality.

	Non-Survivors (Mortality) (*n* = 10)	Survivors (*n* = 122)	*p* Value
Preoperative TSH	1.50 (0.61–2.99)	4.74 (0.03–37.70)	0.006
Postoperative TSH	0.81 (0.09–10.59)	1.69 (0.11–39.00)	0.080
Preoperative FT3	3.06 (2.77–4.61)	2.52 (1.21–6.10)	0.070
Postoperative FT3	2.00 (1.13–2.18)	2.70 (1.23–4.72)	0.001
Preoperative FT4	1.67 (1.02–1.94)	1.54 (0.91–2.17)	0.477
Postoperative FT4	1.45 (0.90–3.23)	1.48 (0.19–2.37)	0.685
Preoperative VIS	7.00 (7.00–7.00)	0.00 (0.00–17.00)	0.002
Postoperative VIS	14.00 (10.00–20.00)	14.00 (0.00–32.00)	0.759
Operation day	13.00 (8.00–29.00)	19.00 (8.00–31.00)	0.191
Aortic cross-clamp time (min)	63.00 (45.00–150.00)	84.00 (0.00–181.00)	0.566
CPB duration (min)	118.00 (80.00–230.00)	131.50 (0.00–300.00)	0.703
Mechanical ventilation duration (days)	22.00 (14.00–36.00)	5.00 (1.00–64.00)	0.000
RACHS score	3.00 (3.00–6.00)	4.00 (2.00–6.00)	0.579

FT4: Free thyroxine, TSH: thyroid-stimulating hormone, FT3: Free triiodothyronine, VIS: Vasoactive Inotropic Score, CPB: Cardiopulmonary Bypass, RACHS: Risk Adjustment for Congenital Heart Surgery.

**Table 7:** Logistic regression analysis for mortality.

	B (β)	SE	OR	95% CI	*p*
RACHS	0.736	0.461	2.09	0.85–5.14	0.110
CPB duration (min)	−0.000	0.006	1.00	0.99–1.01	0.997
Preoperative VIS	0.189	0.111	1.21	0.97–1.50	0.089
Postoperative VIS	0.007	0.079	1.01	0.86–1.18	0.928
Gestational age (weeks)	−0.386	0.269	0.68	0.40–1.15	0.152
Birth weight (g)	0.0002	0.001	1.00	1.00–1.00	0.870
Preoperative TSH	−0.507	0.327	0.60	0.32–1.14	0.121
Postoperative FT3	−1.599	0.778	0.22	0.05–1.03	0.048
Postoperative FT4	0.888	1.014	2.43	0.33–17.73	0.382

FT4: Free thyroxine, TSH: thyroid-stimulating hormone, FT3: Free triiodothyronine, VIS: Vasoactive Inotropic Score, CPB: Cardiopulmonary Bypass, RACHS: Risk Adjustment for Congenital Heart Surgery.

**Table 8:** ROC analysis of postoperative FT3 for predicting mortality.

Area	Asymptotic Sig	Cutt-Off FT3 Level	Sensitivity (%)	Specificity (%)	Asymptotic 95% Confidence Interval	
					Lower Bound	Upper Bound
0.818	<0.01	2.17	72	70	0.732	0.905

ROC: Receiver operating characteristic.

## Data Availability

The data presented in this study are available on request from the corresponding author due to the presence of sensitive patient information, the dataset cannot be made publicly available but can be shared in anonymized form with editors or qualified researchers.

## Abbreviations

The following abbreviations are used in this manuscript:

AUC	Area under the curve
CICU	Cardiac intensive care unit
CPB	Cardiopulmonary bypass
CHD	Congenital heart disease
ECMO	Extracorporeal membrane oxygenation
LCOS	Low cardiac output syndrome
TSH	Thyroid-stimulating hormone
T4	Thyroxine
T3	Triiodothyronine
VIS	Vasoactive inotropic score
